# Discontinuation of adjuvant endocrine therapy and impact on quality of life and functional status in older patients with breast cancer

**DOI:** 10.1007/s10549-022-06583-7

**Published:** 2022-04-19

**Authors:** Annelieke A. Lemij, Nienke A. de Glas, Marloes G. M. Derks, Esther Bastiaannet, Jos W. S. Merkus, Titia E. Lans, Carmen C. van der Pol, Thijs van Dalen, Annelie J. E. Vulink, Leander van Gerven, Onno R. Guicherit, Eugenie M. H. Linthorst-Niers, Frederiek van den Bos, Judith R. Kroep, Gerrit Jan Liefers, Johanneke E. A. Portielje

**Affiliations:** 1 Department of Medical Oncology, Leiden University Medical Center, Leiden, The Netherlands; 2 Department of Surgery, Leiden University Medical Center, P.O. Box 9600, 2300 RC Leiden, The Netherlands; 3 Department of Surgery, Haga Hospital, The Hague, The Netherlands; 4 Department of Surgery, Admiraal de Ruyter Hospital, Goes, The Netherlands; 5 Department of Surgery, Alrijne Hospital, Leiderdorp, Leiden, The Netherlands; 6 Department of Surgery, Diakonessenhuis, Utrecht, The Netherlands; 7 Department of Medical Oncology, Reinier de Graaf Gasthuis, Delft, The Netherlands; 8 Department of Internal Medicine, LangeLand Hospital, Zoetermeer, The Netherlands; 9 Department of Surgery, Haaglanden Medical Center, The Hague, The Netherlands; 10 Department of Surgery, Groene Hart Hospital, Gouda, The Netherlands; 11 Department of Gerontology and Geriatrics, Leiden University Medical Center, Leiden, The Netherlands

**Keywords:** Breast cancer, Endocrine therapy, Older patients, Geriatric assessment

## Abstract

**Purpose:**

Side effects are the main reason for discontinuation of adjuvant endocrine therapy in older adults. The aim of this study was to examine geriatric predictors of treatment discontinuation of adjuvant endocrine therapy within the first 2 years after initiation, and to study the association between early discontinuation and functional status and quality of life (QoL).

**Methods:**

Patients aged ≥ 70 years with stage I–III breast cancer who received adjuvant endocrine therapy were included. The primary endpoint was discontinuation of endocrine therapy within 2 years. Risk factors for discontinuation were assessed using univariate logistic regression models. Linear mixed models were used to assess QoL and functional status over time.

**Results:**

Overall, 258 patients were included, of whom 36% discontinued therapy within 2 years after initiation. No geriatric predictive factors for treatment discontinuation were found. Tumour stage was inversely associated with early discontinuation. Patients who discontinued had a worse breast cancer-specific QoL (*b* = − 4.37; 95% CI − 7.96 to − 0.78; *p* = 0.017) over the first 2 years, in particular on the future perspective subscale (*b* = − 11.10; 95% CI − 18.80 to − 3.40; *p* = 0.005), which did not recover after discontinuation. Treatment discontinuation was not associated with functional improvement.

**Conclusion:**

A large proportion of older patients discontinue adjuvant endocrine treatment within 2 years after initiation, but geriatric characteristics are not predictive of early discontinuation of treatment. Discontinuation of adjuvant endocrine therapy did not positively affect QoL and functional status, which implies that the observed poorer QoL in this group is probably not caused by adverse effects of endocrine therapy.

**Supplementary Information:**

The online version contains supplementary material available at 10.1007/s10549-022-06583-7.

## Introduction

Breast cancer is the most frequently diagnosed malignancy amongst women, with more than 30% of all patients being over 70 years of age at the time of diagnosis [1]. Adjuvant endocrine therapy is a significant part of treatment in patients with high-risk hormone receptor-positive breast cancer because of its beneficial effect on recurrence rates and breast cancer-specific survival [2, 3]. However, whilst the number of patients above 75 years of age receiving endocrine therapy has increased between the years 2000 and 2017, their relative survival rate has not improved [4]. This lack of survival gain might be due to a limited effect of adjuvant endocrine therapy on low-risk early-stage breast cancer in older patients [4, 5]. Another reason might be the higher impact of competing causes of death in older patients [5]. Therefore, other outcomes, such as the impact of therapy on quality of life and functional status merit further exploration [6].

Moreover, despite the recommended minimum of 5 continuous years of adjuvant endocrine therapy, studies show a substantial discontinuation rate within this period of about 40% and ranging from 8 to 73% of patients [7–15]. The main reason for discontinuation is the occurrence of side effects, with a higher proportion of discontinuation in older patients than in younger ones [8, 11–13, 16]. Studies on older patients with breast cancer treated with chemotherapy show a correlation between specific geriatric conditions and toxicity [17, 18]. There is only little information about specific geriatric factors that might contribute to a higher discontinuation rate of endocrine therapy amongst older patients [7]. Therefore, the objective of this study was to investigate adjuvant endocrine therapy discontinuation in older patients with breast cancer, and to analyse geriatric predictive factors for early discontinuation. Another aim was to evaluate whether early discontinuation is associated with changes in functional status and quality of life over time.

## Methods

Patients included in this study were selected from the Climb Every Mountain study (UL-2011-5263). This is a prospective, multicentre observational study. Details of this cohort have been extensively described in previous publications [19, 20]. Briefly, patients were recruited from nine Dutch hospitals between 2013 and 2018 and included women aged ≥ 70 years with primary breast cancer. For this study, patients with hormone receptor-positive breast cancer (ER and/or PR > 10%), stage I–III, who were treated with surgery and adjuvant endocrine therapy were selected. Exclusion criteria were a previous history of breast cancer, distant metastases, the inability to read Dutch and advanced dementia. At baseline, patients underwent a geriatric assessment as part of standard care and follow-up was performed at three, six, twelve and twenty-four months after diagnosis. To obtain as much information as possible on all patients who participated in the CLIMB, including the patients who did not attend for follow-up, information about the tumour characteristics, type of treatment and complications was retrospectively retrieved from the medical records of all patients one year after diagnosis (Supplemental Fig. A). Written informed consent was obtained from all participants and the study was approved by the medical ethics committee of the Leiden University Medical Center.

### Questionnaires

The baseline geriatric assessment included a history of comorbidities prior to breast cancer diagnosis [Charlson comorbidity index (CCI)] [21], use of medication, nutritional status [Malnutrition universal screening tool (MUST)] [22], cognition [Mini mental state examination (MMSE)] [23], physical function [Timed up and go test (TUG)] [24], and functional status using ADL and IADL [Groningen activitiy restriction scale (GARS)] [25]. At follow-up, clinical data including patient, tumour and treatment characteristics with the associated side effects were retrieved from medical records. Tumour stage was classified according to the eighth edition of TNM criteria from the cancer staging manual of the American Joint Committee on Cancer [26]. Follow-up at 3, 6, 12 and 24 months after diagnosis consisted of multiple assessments and questionnaires, including cognition (MMSE), physical function (TUG), functional status (GARS), quality of life (EORTC QLQ-C30 and EORTC QLQ-BR23) [27, 28], the Cantril Ladder for overall patient satisfaction [29]; depression [30]; apathy [31] and loneliness [32]. For breast cancer-specific quality of life, optional questions regarding sexual function, sexual enjoyment and upset by hair loss were excluded from the total score, since these questions were answered by a limited number of patients (Supplemental Fig. B). For the EORTC QLQ-C30, the outcome was assessed as clinically relevant according to the findings from Musoro et al. [33]. For the EORTC QLQ-BR23, a difference of ≥ 10 points was considered to be clinically relevant [34]. The questionnaires from the first follow-up (i.e. three months post-diagnosis) were considered to be the baseline for the analyses of quality of life and the other functional domains, because most patients start adjuvant endocrine therapy around that time.

### Outcome

Discontinuation of the initiated adjuvant endocrine therapy due to toxicity or patient preferences within two years after initiation was defined as the primary outcome for the present study. The golden standard for adjuvant endocrine therapy in postmenopausal women is 2–3 years of tamoxifen followed by 2–3 years of an aromatase inhibitor or 5 years of an aromatase inhibitor [35]. The second choice is 5 years of tamoxifen monotherapy. Therefore, early discontinuation was defined as discontinuation of the initial adjuvant endocrine therapy within two years after start. Changes in quality of life, functional status, life satisfaction, depression, apathy and loneliness over time were assessed as the secondary outcome.

### Statistical analyses

All analyses were performed in IBM SPSS Statistics version 25.0. For all statistical analyses, the threshold for a two-sided, statistically significant p-value was 0.05. All analyses were planned in advance to avoid post hoc analyses. Logistic regression analysis was used to assess predictive factors for discontinuation. We also analysed ‘frailty’, which was defined as impairments in two or more domains: cognition (MMSE < 24), physical function (timed up and go > 12 s), somatic (Charlson comorbidity index ≥ 2 or polypharmacy) or nutrition (high risk on the malnutrition universal screening tool). Patients with a GARS score of ≥ 29 were also considered frail [36]. Linear mixed models were performed to assess longitudinal changes in quality of life, functional status, life satisfaction, depression, apathy and loneliness and whether there were differences in these scores between patients who discontinued therapy and who did not. All outcome measures were seperately analysed as dependent variable with discontinuation and time as fixed parameters. Predefined confounders were also added as fixed parameters to assess the independent effect of adverse events of adjuvant endocrine therapy on these outcome measures. These confounders included age, tumour stage, BMI, Charlson comorbidity index, polypharmacy and type of surgery. Results were presented as beta coefficients (*b*), 95% confidence intervals (CI) and *p*-values.

## Results

Overall, we included 258 patients with hormone receptor-positive breast cancer, stage I–III, who underwent surgery and started on adjuvant endocrine therapy. General characteristics, tumour characteristics and therapies are shown in Table 1. Median age was 74 years old. A fifth of all patients had a Charlson comorbidity index of 2 or higher (17%) prior to breast cancer diagnosis. A total of 95 patients (37%) were ADL/IADL independent and 91 patients (35%) were classified as frail. Most patients had stage I or II disease (84%). Very few patients received chemotherapy either in the neoadjuvant (2%) or adjuvant setting (7%). One hundred twenty-nine patients (50%) started with tamoxifen and 124 patients (48%) with an aromatase inhibitor and it was not specified in 5 patients (2%).

**Table 1:** Patient, tumour and treatment characteristics at baseline

	*N*	%
Age		
70–74	130	50.4
75–79	59	22.9
≥ 80	69	26.7
Charlson comorbidity index (CCI)		
0	146	56.6
1	67	26.0
≥ 2	45	17.4
BMI		
20–24.9	80	31.0
< 20	10	3.9
≥ 25	167	64.7
Unknown	1	0.4
Polypharmacy		
No	155	60.1
Yes	93	36.0
Unknown	10	3.9
Nutritional status (MUST)		
Low risk	224	86.8
Medium risk	9	3.5
High risk	8	3.1
Unknown	17	6.6
Functional status (GARS)		
< 19: no dependency	95	36.8
19–28: some dependency	126	48.8
≥ 29: disabled	35	13.6
Unknown	2	0.8
Cognition (MMSE)		
Normal cognition (≥ 24)	233	90.3
Cognitive impairment (< 24)	9	3.5
Unknown	16	6.2
Physical function (TUG)		
≤ 12 s	164	63.6
> 12 s	50	19.4
Unknown	44	17.0
Current living situation		
Independent	243	94.2
Assisted living	14	5.4
Unknown	1	0.4
Stage		
I	101	39.1
II	116	45.0
III	31	12.0
Unknown	10	3.9
Grade		
I	33	12.8
II	142	55.0
III	75	29.1
Unknown	8	3.1
Hormone receptor status		
ER+/PR+	185	71.7
ER+/PR−	72	27.9
ER−/PR+	1	0.4
HER2		
Negative	201	77.9
Positive	27	10.5
Unknown	30	11.6
Neoadjuvant treatment		
No neoadjuvant treatment	211	81.8
Chemotherapy (CT)	6	2.3
Endocrine therapy (ET)	21	8.1
Combination of ET and CT	0	0.0
Unknown	20	7.8
Most extensive surgery		
Breast conserving	121	46.9
Mastectomy	137	53.1
Most extensive axillary surgery		
No axillary surgery	6	2.3
Sentinel node procedure	183	70.9
Axillary lymph node dissection	66	25.6
Unknown	3	1.2
Adjuvant systemic treatment		
Endocrine therapy (ET)	241	93.4
Combination of ET and CT	17	6.6
Adjuvant radiotherapy		
No	120	46.5
Yes	138	53.5
Adjuvant herceptin (trastuzumab)		
No	251	97.3
Yes	7	2.7

*BMI* body mass index, *MUST* malnutrition universal screening tool, *GARS* groningen activity restriction scale, *MMSE* mini mental state examination, *TUG* timed up and go test, *ER* oestrogen receptor, *PR* progesterone receptor, *HER2* human epidermal growth factor receptor 2

**Fig. 1:**
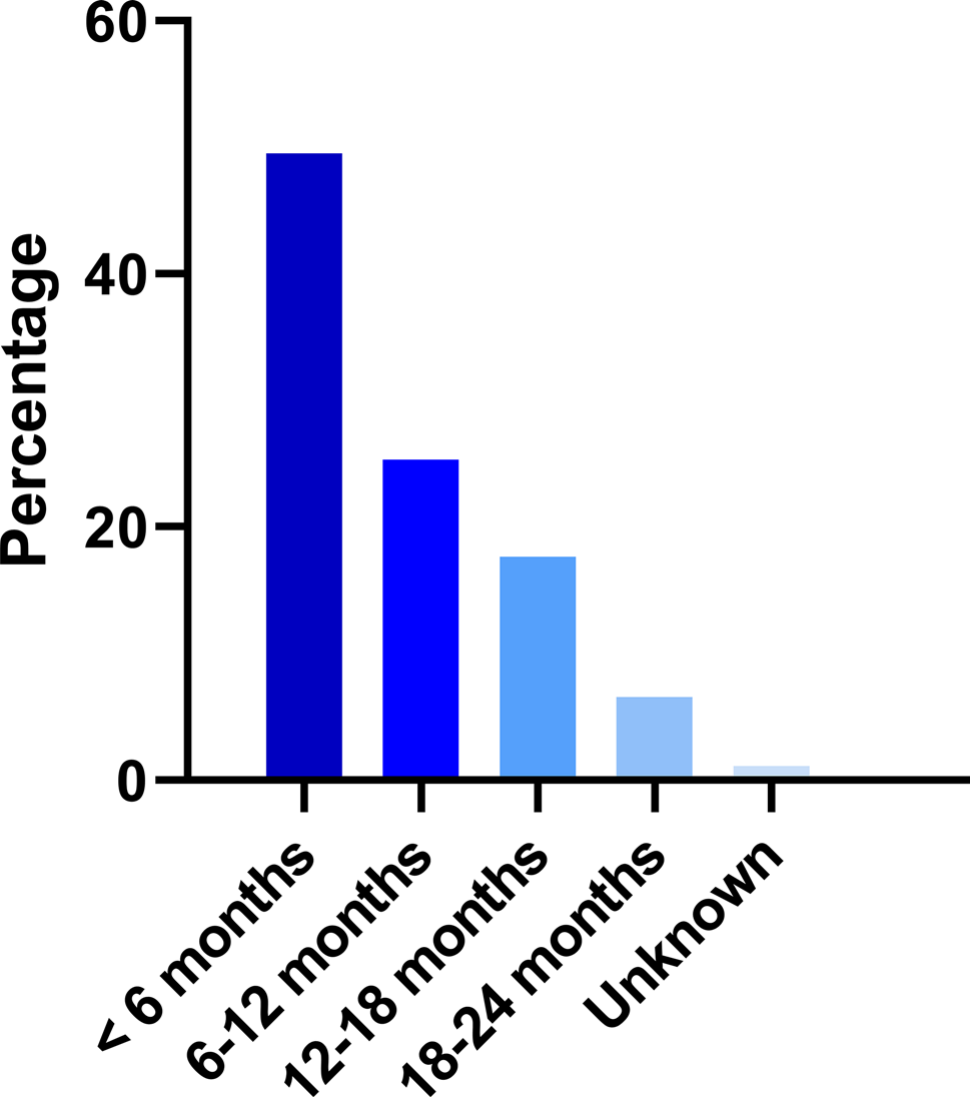
Period of discontinuation of adjuvant endocrine therapy after start

Of patients with adjuvant endocrine therapy, 193 patients (75%) had at least one side effect (Table 2). The most reported side effects were musculoskeletal symptoms in 37% of patients, followed by hot flushes (34%) and fatigue (23%). Some patients experienced severe side effects, such as a thromboembolism (2%), cardiovascular symptoms (2%) or an allergic reaction (2%). In total, 94 patients (36%) discontinued the initiated adjuvant endocrine therapy within 2 years, of which 97% for reasons other than recurrence of breast cancer (Table 2). Half of the patients who discontinued treatment, did so within the first six months and 75% within the first year (Fig. 1). As for the discontinuation rates, there was no statistically significant difference between aromatase inhibitors or tamoxifen.

**Table 2:** Side effects and reason for ﻿discontinuation of adjuvant endocrine therapy within 2 years after initiation

	*N*	%
**Total number of side effects**	434	–
Thromboembolism	5	1.9
Cardiovascular	5	1.9
Allergic reaction	4	1.6
Musculoskeletal	96	37.2
Hot flashes	88	34.1
Fatigue	60	23.3
Psychological	40	15.5
Gastrointestinal	26	10.1
Hair loss and thinning	17	6.6
Vaginal dryness or discharge	13	5.0
Dizziness/balance problems	11	4.3
Dermatological	9	3.5
Other	52	20.2
**At least 1 side effect**	193	74.8
**Discontinuation of endocrine therapy**		
No	164	63.6
Yes	94	36.4
Reasons for early discontinuation		
Recurrence	3	3.2
Toxicity	56	59.6
Not specified	35	37.2

None of the geriatric characteristics or frailty status predicted who would discontinue adjuvant endocrine therapy within two years (Table 3). Patients with a higher tumour stage, however, were less likely to discontinue treatment (stage II: OR 0.42, 95% CI 0.24–0.74, stage III: OR 0.25, 95% CI 0.09–0.65, *p* = 0.001, compared to stage I).

**Table 3:** Association between patient, tumour and treatment characteristics and early discontinuation of adjuvant endocrine therapy < 2 years because of toxicity or non-specified reasons, univariate logistic regression analysis

	Univariate				
	*N* patients (%), total	*N* patients (%), discontinued**	OR	95% CI	*p*-value
Age					0.371
70–74	130 (50.4)	47 (51.6)	Ref		
75–79	59 (22.9)	24 (26.4)	1.21	0.64–2.28	
≥ 80	69 (26.7)	20 (22.0)	0.72	0.38–1.36	
Charlson comorbidity index (CCI)					0.560
0	146 (56.6)	49 (53.8)	Ref		
1	67 (26.0)	23 (25.3)	1.04	0.56–1.91	
≥ 2	45 (17.4)	19 (20.9)	1.45	0.73–2.87	
BMI					0.794
20–24.9	80 (31.0)	29 (31.9)	Ref		
< 20	10 (3.9)	2 (2.2)	0.44	0.09–2.21	
≥ 25	167 (64.7)	60 (65.9)	0.99	0.57–1.72	
Unknown	1 (0.4)	0 (0.0)	*	*	
Polypharmacy					0.600
No	155 (60.1)	56 (61.5)	Ref		
Yes	93 (36.0)	33 (36.3)	0.97	0.57–1.66	
Unknown	10 (3.9)	2 (2.2)	0.44	0.09–2.15	
Nutritional status (MUST)					0.941
Low risk	224 (86.8)	80 (87.9)	Ref		
Medium risk	9 (3.5)	3 (3.3)	0.90	0.22–3.70	
High risk	8 (3.1)	2 (2.2)	0.60	0.12–3.04	
Unknown	17 (6.6)	6 (6.6)	0.98	0.35–2.75	
Functional status (GARS)					0.992
< 19: no dependency	95 (36.8)	33 (36.3)	Ref		
19–28: some dependency	126 (48.8)	46 (50.5)	1.08	0.62–1.89	
≥ 29: disabled	35 (13.6)	12 (13.2)	0.98	0.43–2.22	
Unknown	2 (0.8)	0 (0.0)	*	*	
Cognition (MMSE)					0.764
Normal cognition (≥ 24)	233 (90.3)	81 (89.0)	Ref		
Cognitive impairment (< 24)	9 (3.5)	3 (3.3)	0.94	0.23–3.85	
Unknown	16 (6.2)	7 (7.7)	1.46	0.52–4.06	
Physical function (TUG)					0.030
≤ 12 s	164 (63.6)	66 (72.5)	Ref		
> 12 s	50 (19.4)	17 (18.7)	0.77	0.39–1.49	
Unknown	44 (17.0)	8 (8.8)	0.33	0.14–0.76	
Stage					0.001
I	101 (39.1)	50 (54.9)	Ref		
II	116 (45.0)	34 (37.4)	0.42	0.24–0.74	
III	31 (12.0)	6 (6.6)	0.25	0.09–0.65	
Unknown	10 (3.9)	1 (1.1)	0.11	0.01–0.93	
Grade					0.412
I	33 (12.8)	9 (9.9)	Ref		
II	142 (55.0)	53 (58.2)	1.59	0.69–3.67	
III	75 (29.1)	28 (30.8)	1.59	0.65–3.90	
Unknown	8 (3.1)	1 (1.1)	0.38	0.04–3.55	
Neoadjuvant treatment					0.466
No neoadjuvant treatment	211 (81.8)	78 (85.7)	Ref		
Chemotherapy (CT)	6 (2.3)	2 (2.2)	0.85	0.15–4.76	
Endocrine therapy (ET)	21 (8.1)	4 (4.4)	0.40	0.13–1.24	
Unknown	20 (7.8)	7 (7.7)	0.92	0.35–2.40	
Most extensive surgery					0.165
Breast conserving	121 (46.9)	48 (52.7)	Ref		
Mastectomy	137 (53.1)	43 (47.3)	0.70	0.42–1.16	
Adjuvant systemic treatment					0.129
Endocrine therapy (ET)	241 (93.4)	88 (96.7)	Ref		
Combination of ET and CT	17 (6.6)	3 (3.3)	0.37	0.10–1.33	
Frailty					0.746
No	167 (64.7)	74 (81.3)	Ref		
Yes	91 (35.3)	17 (18.7)	0.90	0.47–1.72	

*OR* odds ratio, *95% CI* 95% confidence interval, *BMI* body mass index, *MUST* malnutrition universal screening tool, *GARS* groningen activity restriction scale, *MMSE* mini mental state examination, *TUG* timed up and go test

*Could not be calculated because of the small numbers

**Discontinuation of adjuvant endocrine therapy for reasons other than recurrence

**Fig. 2:**
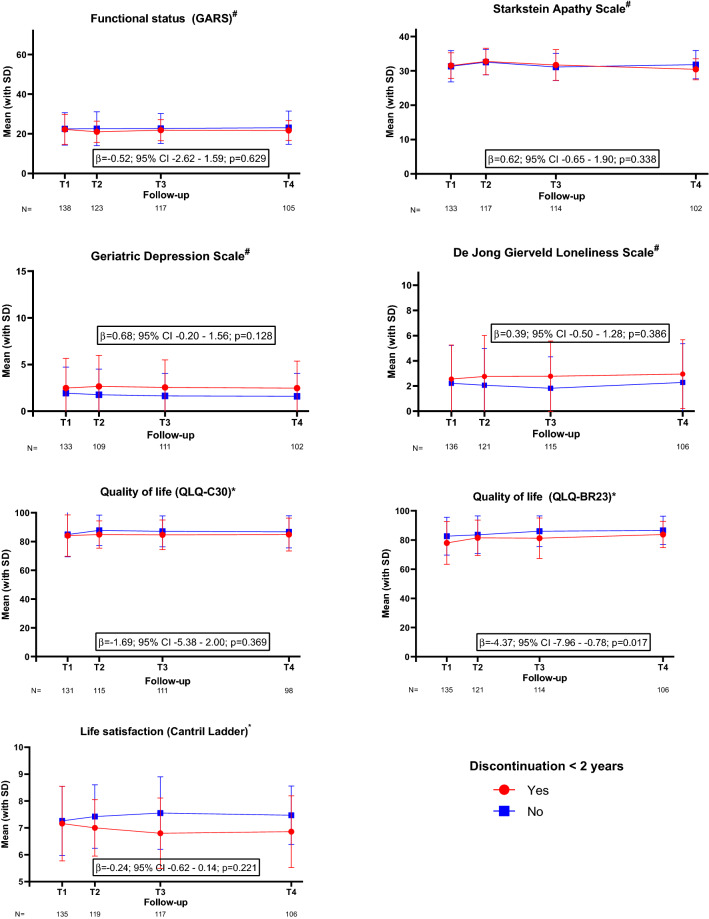
Functional status, apathy, depression, loneliness, general quality of life, breast cancer-specific quality of life and life satisfaction over time. #A higher score indicates a worse outcome; *A higher score indicates a better outcome. Adjusted for age, tumour stage, BMI, Charlson comorbidity index, polypharmacy and type of surgery.T1—baseline, 3 months after diagnosis, start adjuvant endocrine therapy; T2—6 months after diagnosis; T3—12 months after diagnosis; T4—24 months after diagnosis

**Fig. 3:**
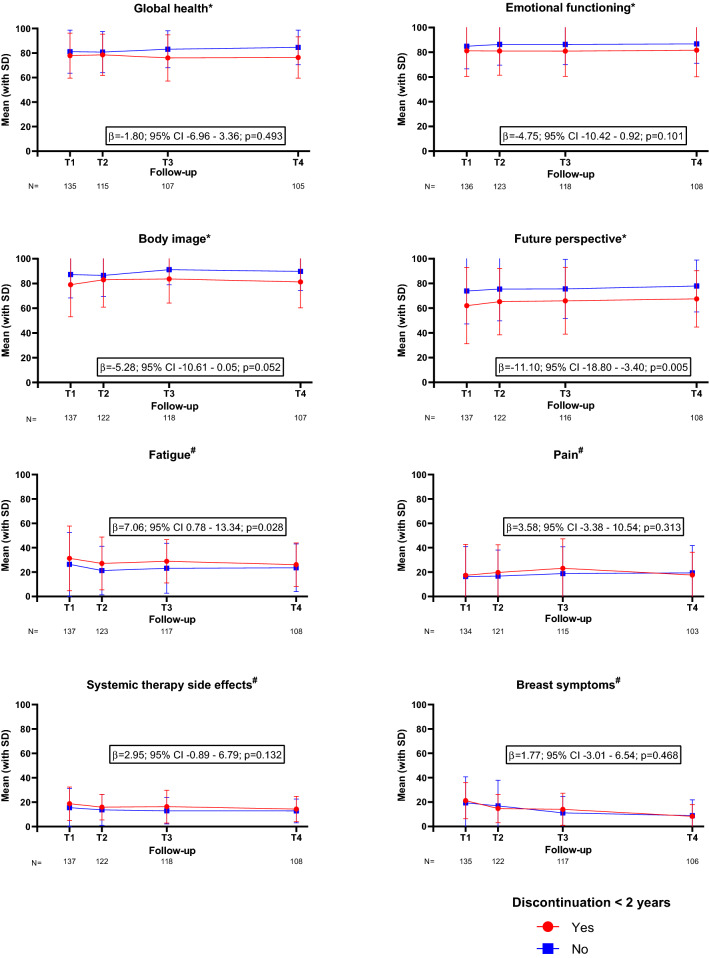
Selection of subscales from the EORTC QLQ-C30 and QLQ-BR23 quality of life questionnaires. #A higher score indicates a worse outcome; *A higher score indicates a better outcome. Adjusted for age, tumour stage, BMI, Charlson comorbidity index, polypharmacy and type of surgery. T1—baseline, 3 months after diagnosis, start adjuvant endocrine therapy; T2—6 months after diagnosis; T3—12 months after diagnosis; T4—24 months after diagnosis

One hundred sixty-five patients (64%) participated in the follow-up questionnaires (Supplemental Fig. A). After adjustment for predefined confounders, patients who discontinued endocrine therapy within two years had a longitudinal clinically relevant reduction in breast cancer-specific quality of life in the first 24 months post-diagnosis (*b* = − 4.37; 95% CI − 7.96 to − 0.78; *p* = 0.017, Fig. 2), in particular on the future perspective subscale (*b* = − 11.10; 95% CI − 18.80 to − 3.40; *p* = 0.005, Fig. 3). These patients also showed worse scores on the fatigue subscale (*b* = 7.06; 95% CI 0.78–13.34; *p* = 0.028, Fig. 3). As for the functional status, life satisfaction, depression, apathy and loneliness, there was no statistical difference between patients who discontinued therapy and those who continued (Fig. 2).

## Discussion

During the first two years of treatment, a relatively high proportion of older patients discontinued the initiated adjuvant endocrine therapy, with the majority of patients stopping within the first six months. A higher tumour stage was inversely associated with discontinuation. No geriatric predictive factors for treatment discontinuation were found. Regarding the quality of life, patients who discontinued treatment for other reasons than recurrence or death had clinically relevant worse scores on future perspective and fatigue subscales, but these did not recover after discontinuation, suggesting that this lower score is not related to possible side effects of endocrine treatment itself. Other domains were not statistically significantly different in patients who discontinued adjuvant endocrine therapy compared to those who did continue therapy in the first two years after diagnosis.

This study was not able to find any geriatric factors that were associated with early adjuvant endocrine therapy discontinuation. Previous studies showed that cognition, frailty status and poor sleep quality were associated with poor adherence to adjuvant endocrine therapy [10, 11]. Cognition was also tested in this study, but the small number of patients with cognitive impairment included, prevents reliable determination of an association. In this study, patients with unfavourable tumour characteristics were less likely to discontinue treatment. Other studies have also explored the association between tumour stage and discontinuation of adjuvant endocrine therapy, but the results have been inconsistent [7, 10–12]. A study of Bluethmann et al. including 1000 patients aged ≥ 65 years with stage I–IIIa breast cancer, showed that patients with a higher stage had a lower hazard ratio compared to stage I for early and late discontinuation of adjuvant endocrine therapy. However, Kidwell et al. with 500 postmenopausal patients of 35–89 years of age (median age 59) with stage 0–III breast cancer, did not find an association between stage and early discontinuation of adjuvant endocrine therapy [10]. It might be possible that this relation is only evident in older patients.

Moreover, the association between discontinuation and tumour stage may implicate that motivation and oncologist’s recommendations play a major role in continuation of treatment. This hypothesis is supported by the results of Fink et al. showing that patients with neutral or negative beliefs about risks and benefits of therapy were more likely to discontinue treatment early [14]. Furthermore, a study by Sheppard et al. although tested in a limited number of patients, showed that less optimistic patients were more likely to discontinue therapy than those who were more optimistic [12]. This seems to be in line with other studies showing that optimism is related to improved health outcomes, with optimists being better at taking health conductive action because of a greater sense of projecting oneself into the future and making a judgement that things will be good [37]. This assumption concurs with the current study in which patients that continued therapy had a better score on the future perspective scale. Similar results were seen for the fatigue subscale and breast cancer-specific quality of life. A previous study found a similar worse breast cancer-specific quality of life in patients who discontinue therapy compared to those who continue therapy both at baseline and during follow-up in patients of all age groups [38]. The role of medication beliefs and illness perceptions (i.e. views, ideas, cognitions and emotions a patient has about the disease) is currently being investigated in the ADHERE trial (NL8541).

This aspect underlines the importance of the role of the physician in explaining about the balance of benefits and risks of therapy and that incorporating interventions into clinical practice to promote treatment continuation is critical for sustaining. An important consideration of this risk-benefit ratio in older patients is that the beneficial effect of adjuvant treatment might differ from younger patients due to competing risk of mortality [39]. Interestingly, a study investigating persuasion in decision-making about adjuvant treatment in patients with breast cancer, showed that at higher tumour stages oncologists were more likely to steer towards intensifying adjuvant chemotherapy [40]. However, tumour stage did not affect persuasive behaviours of oncologists for endocrine therapy. Nevertheless, the current study shows that in the occurrence of side effects patients with higher tumour stages are more likely to continue adjuvant endocrine therapy, which is probably due to motivational interviewing. Therefore, motivational interviewing in this group of patients might improve persistence of adjuvant endocrine therapy.

Strengths of this study include the prospective design with detailed information about a large number of older patients on baseline and follow-up. There are also several limitations to our study. First, this study was not primarily designed to collect detailed information about treatment discontinuation. However, it was a planned analysis in which the reason for discontinuation was extracted from medical records, which did not always contain specific reasons. Moreover, the questionnaires were only completed at prespecified time points, making it more difficult to determine the direct effect of treatment discontinuation on certain outcome measures. However, in the current study, we showed that even after discontinuation patients still had a statistically significant worse quality of life, which implies that this worse quality of life is not due to endocrine therapy. Another disadvantage of retrieving information from medical records in the first two years is that it might result in underrepresentation of discontinuation rates. However, the reported rate of the current study is in line with previous research and this study showed that most patients discontinued therapy within the first six months after initiation [9–13]. Of note, in the study by Hershman et al. the incidence of treatment discontinuation of aromatase inhibitors progressively increased from year 1 to year 4 in patients of all age groups, whilst discontinuation rates of tamoximen decreased over time [13]. In the current study, we did not find such a difference between early discontinuation of aromatase inhibitors and tamoxifen. This difference might be explained by the fact that Hershman et al. deducted discontinuation rates from prescriptions, in which they had to make several assumptions. They were also unable to determine the reason of discontinuation.

In conclusion, this study illustrates that a large proportion of older patients with breast cancer discontinues adjuvant endocrine therapy within the first two years after initiation. None of the geriatric factors that we explored predicted the rate of early discontinuation. A higher tumour stage was inversely associated with discontinuation. Patients who discontinue early had a worse breast cancer-specific quality of life and worse scores on fatigue and future perspective subscales. Following their discontinuation of adjuvant therapy, these scores did not improve, which implies that the poorer quality of life is probably not caused by adverse effects of endocrine therapy. Future studies should investigate strategies to motivate patients to continue adjuvant endocrine therapy, especially when the benefits outweigh the risks.

## Supplementary Information

Below is the link to the electronic supplementary material.

Supplementary file1 (PDF 370 kb)

## Data Availability

The dataset generated during and/or analysed during the current study are not publicly available due to participant privacy but are available from the corresponding author on reasonable request.
