# Supplementary material for: Discontinuation of adjuvant endocrine therapy and impact on quality of life and functional status in older patients with breast cancer
Source: Breast Cancer Res Treat. 2022 Apr 19;193(3):567–77. doi: 10.1007/s10549-022-06583-7 (PMC9114046; doi:10.1007/s10549-022-06583-7)
Supplement: Supplementary file 1 — Supplementary file1 (PDF 370 kb) [file 10549_2022_6583_MOESM1_ESM.pdf]

Discontinuation of adjuvant endocrine therapy and impact on quality of life and functional status in older patients with breast cancer

Annelieke A. Lemij[a,b], Nienke A. de Glas[a], Marloes G.M. Derks[a], Esther Bastiaannet[a], Jos W.S. Merkus[c], Titia E. Lans[d], Carmen C. van der Pol[e], Thijs van Dalen[f], Annelie J.E. Vulink[g], Leander van Gerven[h], Onno R. Guicherit[i], Eugenie M.H. Linthorst-Niers[j], Frederiek van den Bos[k], Judith R. Kroep[a], Gerrit-Jan Liefers[b], Johanneke E.A. Portielje[a]

[a]Department of Medical Oncology, Leiden University Medical Center, Leiden, The Netherlands

[b]Department of Surgery, Leiden University Medical Center, Leiden, The Netherlands

[c]Department of Surgery, Haga Hospital, The Hague, The Netherlands

[d]Department of Surgery, Admiraal de Ruyter Hospital, Goes, The Netherlands

[e]Department of Surgery, Alrijne Hospital, Leiden and Leiderdorp, The Netherlands

[f]Department of Surgery, Diakonessenhuis, Utrecht, The Netherlands

[g]Department of Medical Oncology, Reinier de Graaf Gasthuis, Delft, The Netherlands

[h]Department of Internal Medicine, LangeLand Hospital, Zoetermeer, The Netherlands

[i]Department of Surgery, Haaglanden Medical Center, The Hague, The Netherlands

[j]Department of Surgery, Groene Hart Hospital, Gouda, The Netherlands

[k]Department of Gerontology & Geriatrics, Leiden University Medical Center, Leiden, The Netherlands

Corresponding author

G.J. Liefers

Leiden University Medical Center, department of Surgery

P.O. Box 9600

2300 RC Leiden

The Netherlands

+31 71 526 40 05

g.j.liefers@lumc.nl

Breast Cancer Research and Treatment

Supplemental figure A: Flowchart

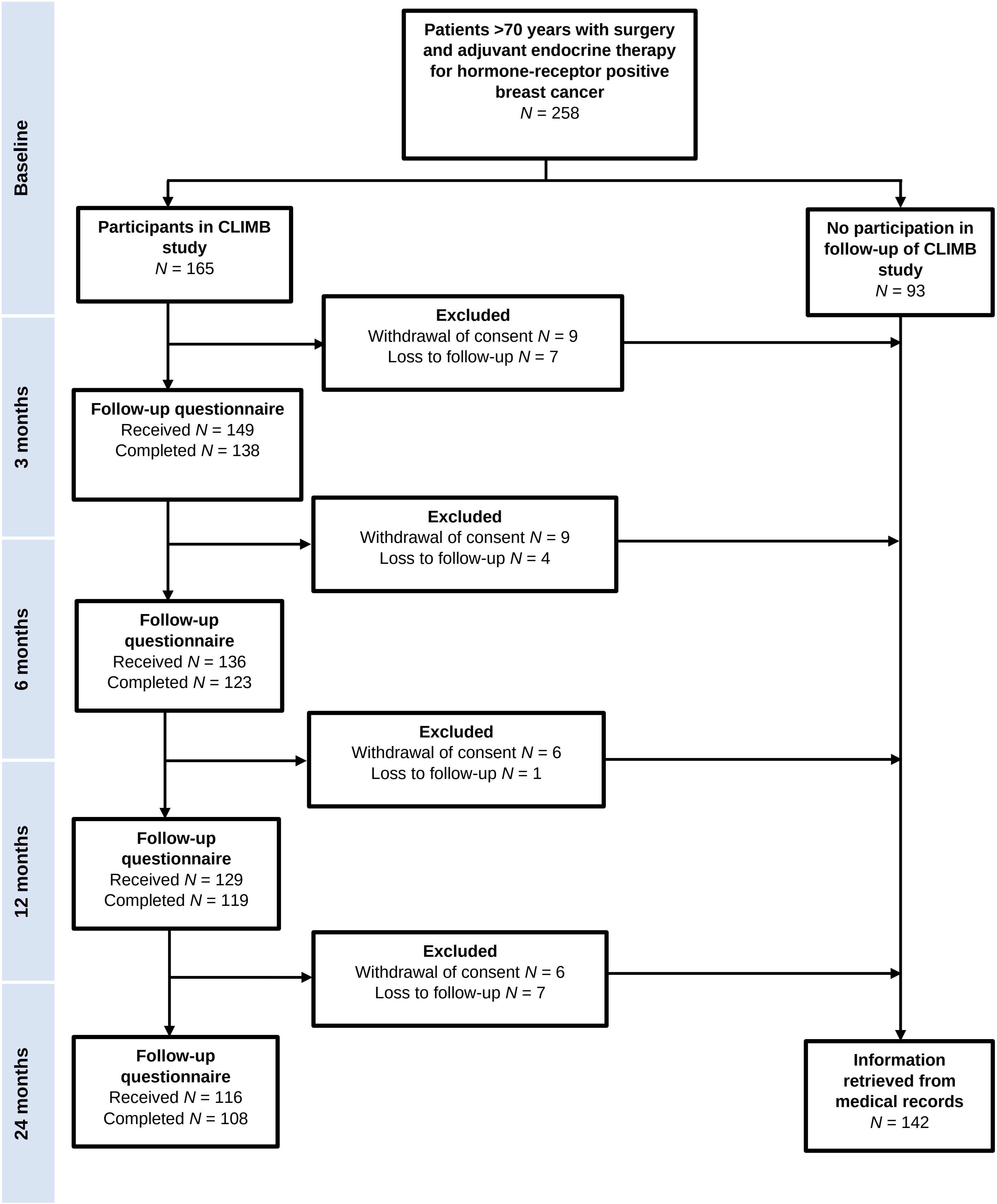

Supplemental figure B: Other subscales from the EORTC QLQ30 and QLQ BR23 questionnaires

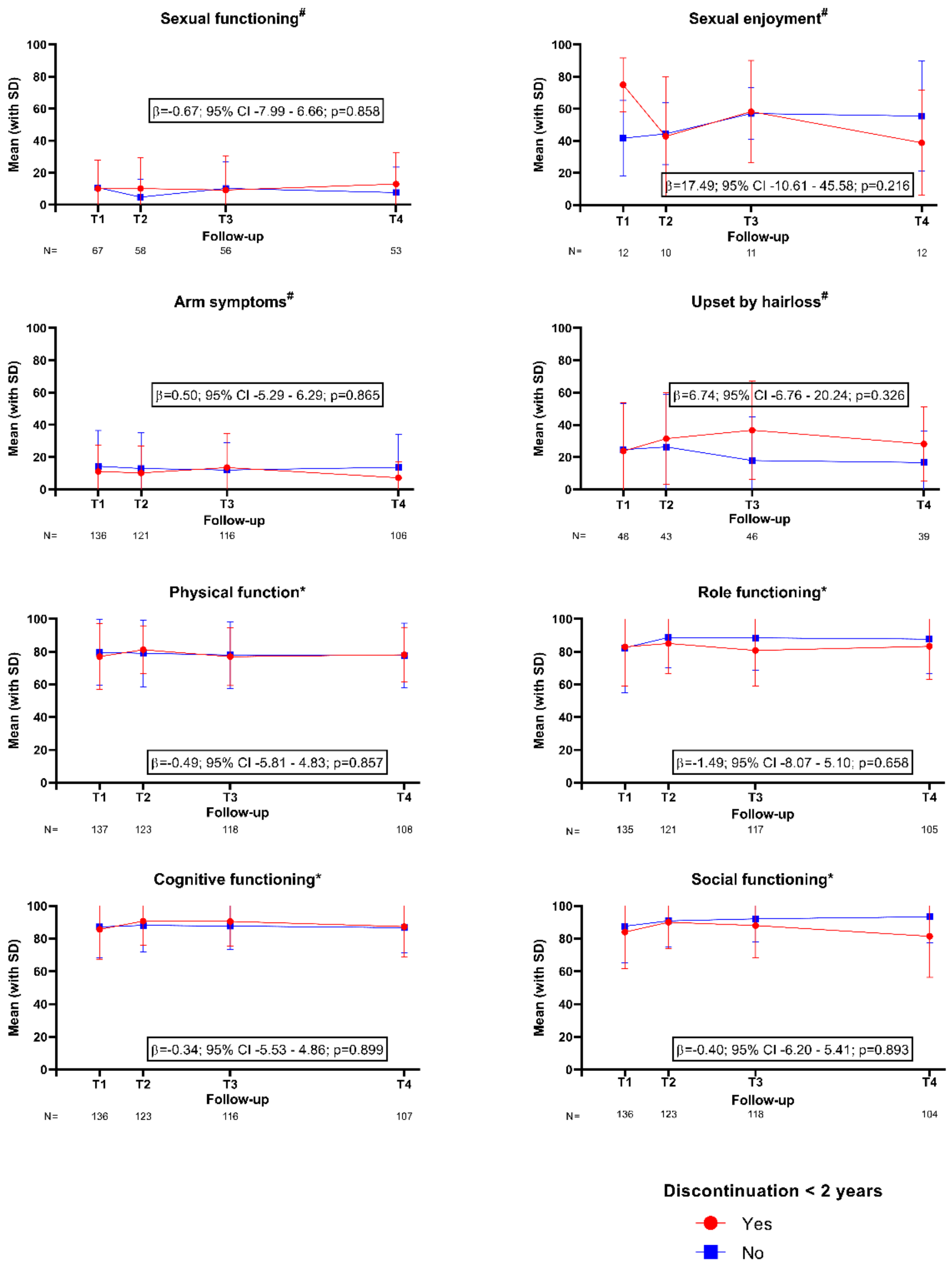

*#A higher score indicates a worse outcome; *A higher score indicates a better outcome.*
Adjusted for age, tumour stage, BMI, Charlson Comorbidity Index, polypharmacy, and type of surgery.
T1 – baseline, 3 months after diagnosis, start adjuvant endocrine therapy; T2 – 6 months after diagnosis; T3 – 12 months

Supplemental figure C: Other subscales from the EORTC QLQ30 and QLQ BR23 quality of life questionnaires

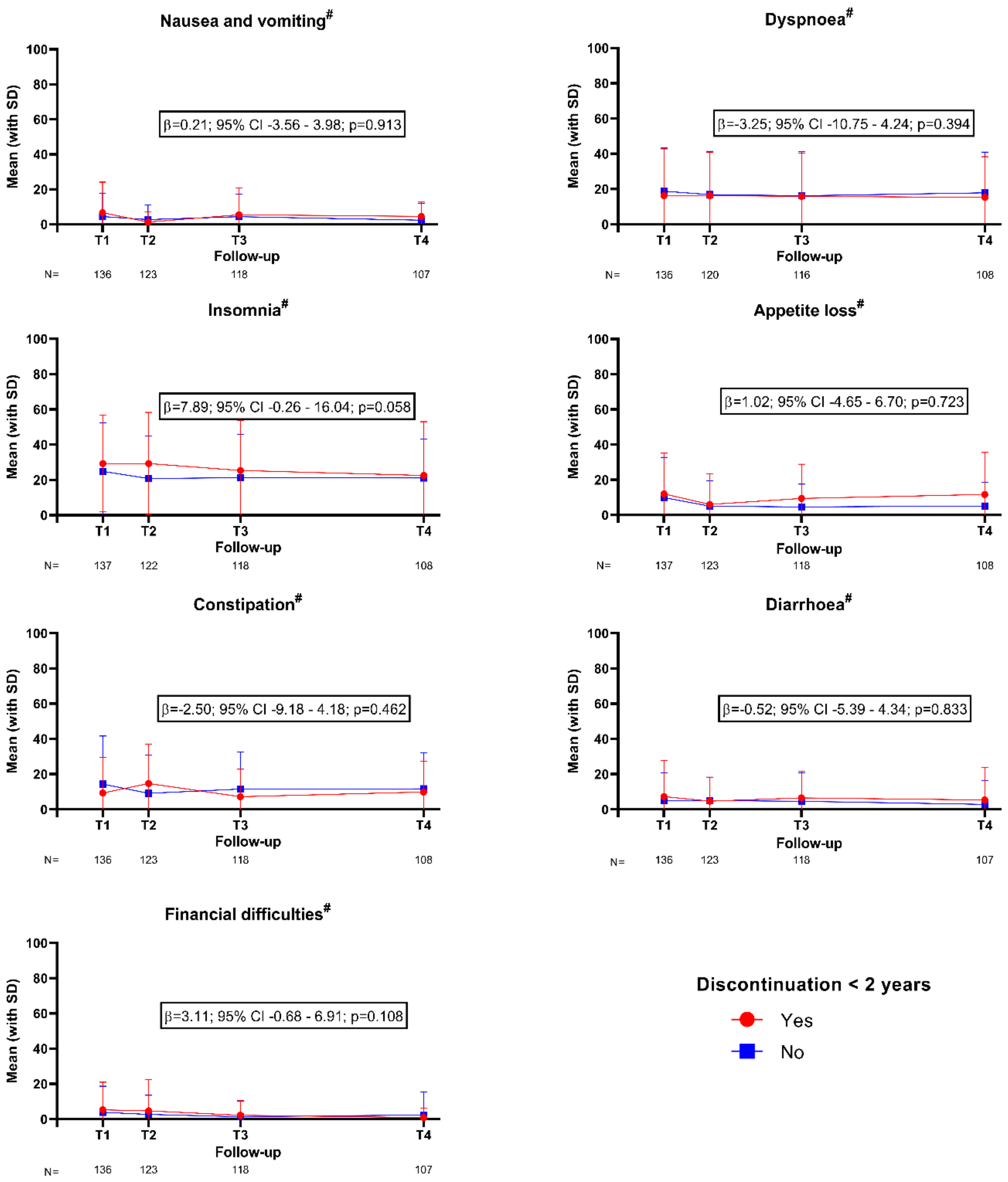

*#A higher score indicates a worse outcome; *A higher score indicates a better outcome.*
Adjusted for age, tumour stage, BMI, Charlson Comorbidity Index, polypharmacy, and type of surgery.
T1 – baseline, 3 months after diagnosis, start adjuvant endocrine therapy; T2 – 6 months after diagnosis; T3 – 12 months after diagnosis; T4 – 24 months after diagnosis
